# In Vitro Antiviral Activity of Tyrosinase from Mushroom *Agaricus bisporus* against Hepatitis C Virus

**DOI:** 10.3390/ph14080759

**Published:** 2021-08-03

**Authors:** David Lopez-Tejedor, Rafael Claveria-Gimeno, Adrian Velazquez-Campoy, Olga Abian, Jose M. Palomo

**Affiliations:** 1 Department of Biocatalysis, Institute of Catalysis (CSIC), c/Marie Curie 2, Cantoblanco Campus UAM, 28049 Madrid, Spain; dlopez@csic.es; 2 Institute of Biocomputation and Physics of Complex Systems (BIFI), Joint Unit IQFR-CSIC-BIFI, Universidad de Zaragoza, 50018 Zaragoza, Spain; rafacg@bifi.es; 3 Fundación Instituto de Investigación Sanitaria de Aragón (IIS Aragón), 50009 Zaragoza, Spain; adrianvc@unizar.es; 4 Centro de Investigación Biomédica en Red en el Área Temática de Enfermedades Hepáticas y Digestivas (CIBERehd), 28029 Madrid, Spain; 5 Departamento de Bioquímica y Biología Molecular y Celular, Universidad de Zaragoza, 50009 Zaragoza, Spain; 6 Instituto Aragonés de Ciencias de la Salud (IACS), 50009 Zaragoza, Spain; 7 Fundación ARAID, Gobierno de Aragón, 50018 Zaragoza, Spain

**Keywords:** hepatitis C virus, tyrosinase, antiviral agent, pharmaceuticals, enzymatic activity, oxidation

## Abstract

Tyrosinases from a commercial *Agaricus bisporus* protein extract and directly isolated from white mushrooms were purified in order to obtaining the well-known tyrosinase from *A. bisporus* (*TyrAB*) of 45 kDa and a newly discovered 50 kDa tyrosinase isoform (*Tyr50 kDa*), and tested showing high antiviral activity against the hepatitis C virus for the first time. Cell toxicity and antiviral activity of tyrosinases were determined in cultured Huh 5-2 liver tumor cells transfected with a replicon system (a plasmid that includes all non-structural hepatitis C virus proteins and replicates autonomously). *TyrAB* was able to inhibit the replication of the hepatitis C virus without inducing toxicity in liver cells. In addition, the post-translational isoform *Tyr50 kDa* showed higher antiviral capacity than the former (up to 10 times greater), also exhibiting 10 times higher activity than the commercial drug Ribavirin^®^. This antiviral activity was directly proportional to the enzymatic activity of tyrosinases, as no antiviral capacity was observed in the inactive form of the enzymes. The tyrosinases approach could represent a new antiviral inhibition mechanism, through a plausible catalytic mechanism of selective hydroxylation of the key role of tyrosine residues in viral proteases.

## 1. Introduction

**Figure 1:**
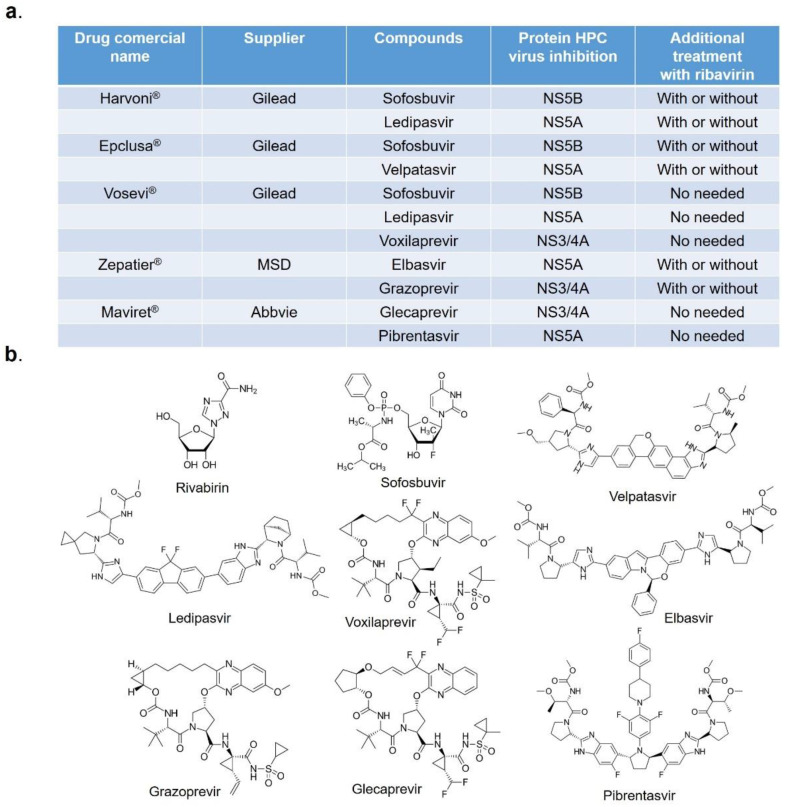
Currently marketed drugs against hepatitis C virus. (**a**) Pharmacological preparations. (**b**) Chemical structures of active molecules.

Infection by hepatitis C virus (HCV) is a main health problem worldwide [1]. HCV contains a small, single-stranded positive RNA that encodes for several proteins that are translated as a large polyprotein, which is processed in the endoplasmic reticulum of the infected cell by viral and host proteases in order to render the individual structural and non-structural viral proteins functional [2]. Among these, the non-structural proteins [3,4] NS2, NS3/4A, NS4B, NS5A, and NS5B present great importance, because most of the drugs currently marketed with therapeutic indication (Figure 1) act through inhibition mechanisms directed towards one of these proteins [5,6,7,8,9].

NS2 is for assembly and replication thanks to its function as an autocatalytic cysteine protease [10]. The N-terminal domain of the multifunctional NS3/4A (or just NS3) is the second viral protease responsible for processing most of the viral polypeptide, while the C-terminal domain of NS3 possesses a helicase/dNTPase function [11]. NS4A is a small hydrophobic protein that serves as a cofactor for enhancing the activity of the NS3 serine protease [12]. NS4B and NS5A are proteins involved in replication and assembly [13], and NS5B is the viral RNA-dependent RNA polymerase that forms a replication complex together with all other non-structural proteins [14].

Currently, some of the best-known systems in the formulation of drugs against HCV consist of a mixture of several molecules (Figure 1) [15]. For example, Harvoni^®^, the last drug registered by Gilead in 2014, is a combination of sofosbuvir and ledipasvir, inhibitory compounds against NS5B and NS5A proteins, respectively (Figure 1). Other commercial drugs consisting of a combination of compounds are the following: Epclusa^®^ (Gilead), Vosevi^®^ (Gilead), Zepatier^®^ (MSD), and Maviret^®^ (Abbvie) (Figure 1) [15].

There are chemical compounds, natural and synthetic, with antiviral capacity against HCV, for example, sesquiterpenoids extracted from the fermentation of the Trichoderma harzianum fungus [16], peptide compounds [17], GSK3082 analogues [18], or aromatic drugs mimetics [19].

However, currently available treatments for the hepatitis C virus are too expensive and many patients, especially from less developed countries, do not have access to them. They are treatments that cost around €60,000 [20], thus, even in developed countries, health budgets are exceeded, forcing governments to establish restrictive criteria for the prescription of these drugs. With a gross estimation of 150–200 million people infected by HCV worldwide, it is necessary and urgent to develop effective and alternative treatments to the existing ones that are also inexpensive.

Tyrosinases are copper-metalloenzymes with a key role in catalytic biological systems [21]. This is an important enzyme in controlling the formation of melanin in melanosome, and plays a key role in the pigmentation of hair and skin. The abnormal expression or activation of tyrosinase is associated with several diseases such as albinism, vitiligo, melanoma, and Parkinson disease [22,23,24]. In particular, tyrosinase from common white button mushrooms *Agaricus bisporus* (*TyrAB*), with broad availability compared with human tyrosinase, has been used in the development of TYR–inhibitors compounds [25]. Recently, it has been described that novel functions or novel activities of some part of them, especially light subunit Orf239342, are very important in cancer proliferation inhibition [26,27,28,29].

In this work, we report for the first time the high inhibition capability against HCV of a tyrosinase and one isoform of it present in the white mushrooms (*Agaricus bisporus*). This presents an HCV inhibition mechanism different from those currently described (allosteric or orthosteric inhibition of non-structural HCV proteins).

## 2. Results and Discussion

**Figure 2:**
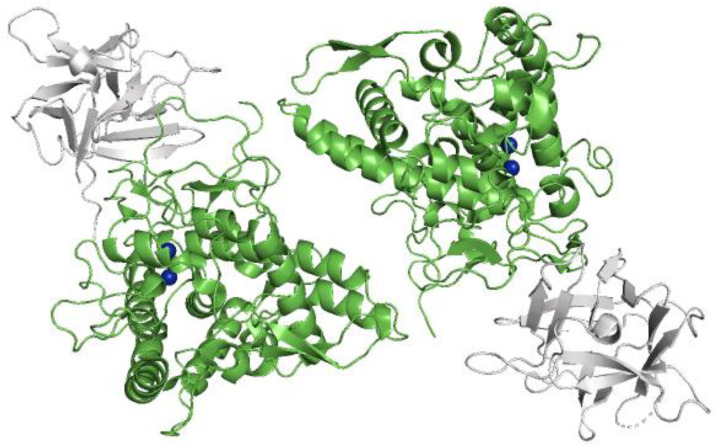
Three-dimensional structure of *Agaricus bisporus* tyrosinase *TyrAB*. H subunits (green), L subunits (grey). Copper atoms (blue). Structure was obtained from PDB data bank with the following codes: 2Y9W. Figure was drawn using Pymol program.

First, both toxicity and antiviral activity of the commercial *Agaricus bisporus* tyrosinase extract against HCV in Huh-5-2 cells were evaluated. The known *Agaricus bisporus* tyrosinase (*TyrAB*) is a hetero-tetramer (in fact, a dimer of heterodimers) made up of two H_1_L_1_H_2_L_2_ subunits, with two 45 kDa high (H) subunits and two 12 kDa light (L) subunits (Figure 2) [25]. Moreover, this extract contains another protein, which we recently founded as a tyrosinase isoform of 50 kDa (*Tyr50 kDa*) [30]. This commercial extract is partially purified (Figure S1).

**Figure 3:**
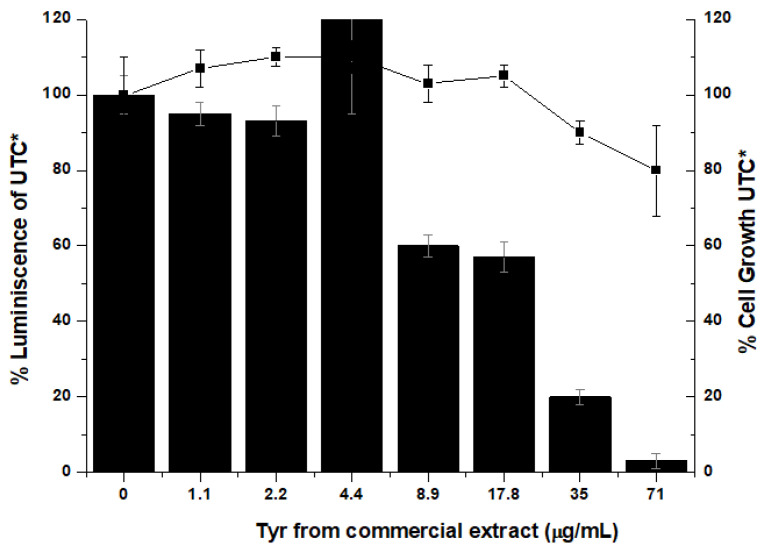
Inhibition of the hepatitis C virus (HCV) replicon in cell assays. Evaluation of the potency and cytotoxicity of the protein in cell (Huh-5-2) assays. HCV replicon replication rate (luminescence, black bars) and cell survival (closed squares) were independently measured in cell culture by increasing protein concentration to determine EC_50_. * All luminescence and viability values were normalized considering the untreated control values (UTC).

This commercial protein solution containing both tyrosinases was analyzed at two pH values (5 and 7) in order to evaluate their cells’ toxicity. In both cases, the solution did not show substantial toxicity towards liver cells (Figure 3). Moreover, cytotoxicity against HeLa cells was evaluated and no cytotoxicity was found (data not shown). However, the solution showed an unexpected inhibition activity of virus replication, with an EC_50_ value of 20 μg/mL and with almost complete inhibition at concentrations of 71 μg/mL (Figure 3). Thus, in order to demonstrate that this viral inhibition activity depends on the tyrosinase, this extract solution of *Agaricus bisporus* tyrosinases was purified.

For that, the commercial solution was purified by direct adsorption on an octadecyl-Sepabeads support in 100 mM buffer pH 7 solution following our previously described protocol [30]. This was based on the suitable hydrophobicity of the tyrosinase surface (Figure S2). Both tyrosinases were immobilized on this solid-support, confirmed by SDS-PAGE (Figure S3), then they were recovered in soluble form through the use of Triton X-100 0.25% (*w*/*v*). A step to reduce the final amount of detergent on the solution was then attempted (for a final concentration of 0.03%). The corresponding purified protein solution containing different detergent concentrations was evaluated against cells.

**Figure 4:**
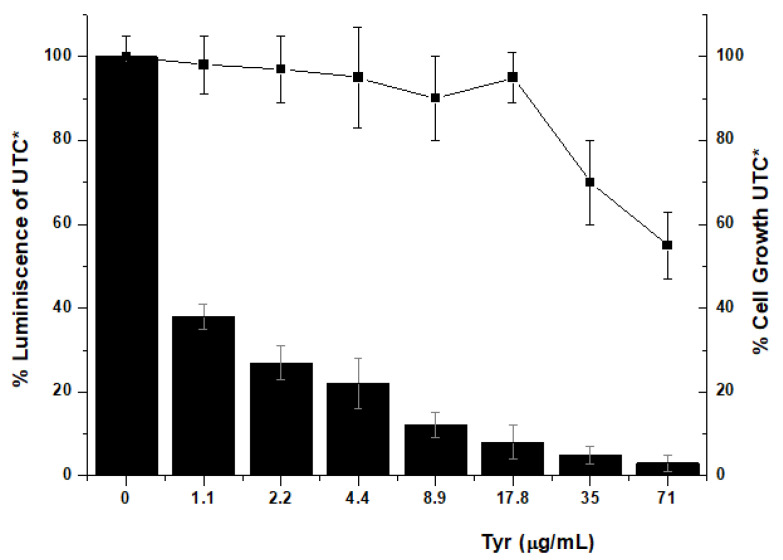
Inhibition of the hepatitis C virus (HCV) replicon in cell assays using a purified protein sample containing both TyrAB and *Tyr50 kDa*. Evaluation of the potency and cytotoxicity of the protein in cell (Huh-5-2) assays. HCV replicon replication rate (luminescence, black bars) and cell survival (closed squares) were independently measured in cell culture by increasing protein concentration to determine EC_50_. * All luminescence and viability values were normalized considering the untreated control values (UTC).

We observed that Triton X-100 concentration of 0.25% (*v*/*v*) clearly affected to the assay (data not shown), the known effect of non-anionic detergent to create able membrane pores, and the final disaggregation of cellular membranes. However, the purified sample (both tyrosinases) containing 0.03% of detergent gave rise to an even better outcome: slight cellular toxicity and an EC_50_ of 1 μg/mL (Figure 4).

This demonstrated that the active tyrosinases were responsible for the antiviral activity of the protein sample.

To further confirm these results and to assess which tyrosinase variant was mainly responsible for the antiviral activity, both enzymes (*TyrAB* and *Tyr50 kDa*) present in the extract were isolated as pure form using the recently described protocol [30]. The different hydrophobicity between *TyrAB* and *Tyr50 kDa* [30] was used for purification. Adsorption on octadecyl-Sepabeads at high ionic strength and in the presence of 0.07% detergent allowed to obtain pure *Tyr50 kDa* protein in the supernatant and complete *TyrAB* adsorbed on the solid support (Figure S4).

**Figure 5:**
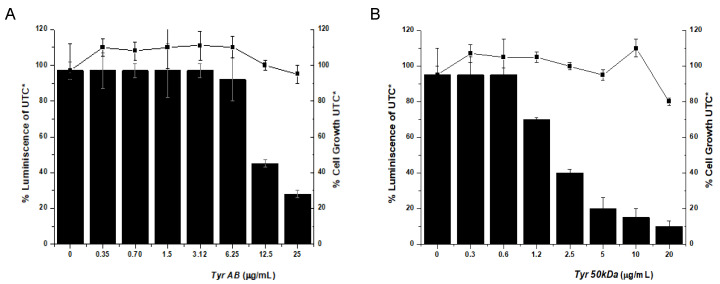
Inhibition of the hepatitis C virus (HCV) replicon in cell assays. Evaluation of the potency and cytotoxicity of pure isoforms in cell (Huh-5-2) assays. HCV replicon replication rate (luminescence, black bars) and cell survival (closed squares) were independently measured in cell culture by increasing protein concentration to determine EC_50_ for (**A**) purified TyrAB and (**B**) purified Tyr50 kDa. * All luminescence and viability values were normalized considering the untreated control values (UTC).

No cellular toxicity was observed for any of the enzymes, and both enzymes showed antiviral activity, although the *Tyr50 kDa* isoform was responsible for most of the activity, with an inhibition capability of 4–10 times compared with that observed for *TyrAB* (EC_50_ of 1.2–2.5 μg/mL for *Tyr50 kDa* compared with 6–12 µg/mL for *TyrAB*) (Figure 5, Table 1).

This result indicated that tyrosinase from mushroom exhibits inhibitory activity against HCV, but also revealed that the isolated isoform (*Tyr50 kDa*)—recently discovered in our laboratory—was responsible for most of the antiviral activity. Analysis and N-terminal protein sequencing of *Tyr50 kDa* (protein sequence exactly matches that of *TyrAB*) suggested that it corresponds to a post-translationally modified isoform of *TyrAB*, probably by glycosylation [30].

**Figure 6:**
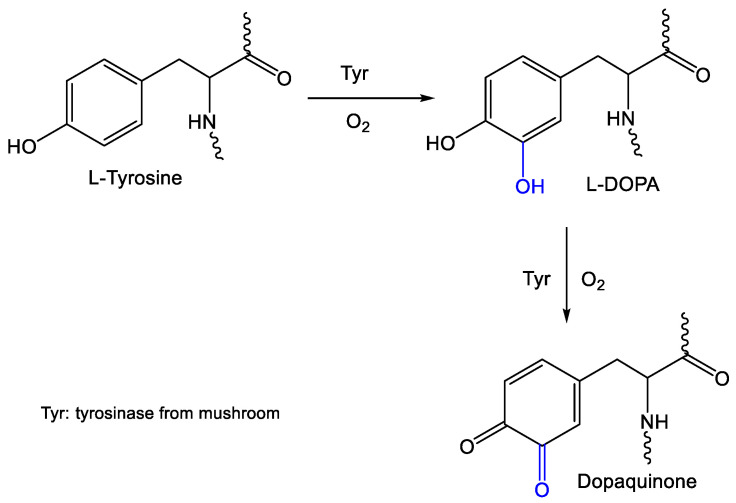
Proposal enzymatic activity capacity of tyrosinase from mushroom *A. bisporus* by selective oxidation of tyrosine residues.

These results provide evidence for a mechanism for inhibiting viral replication based on an enzymatic activity, in this case, based on the selective oxidation of L-tyrosine to L-3,4-dihydroxy-phenylalanine (L-DOPA) and dopaquinone (Figure 6).

**Figure 7:**
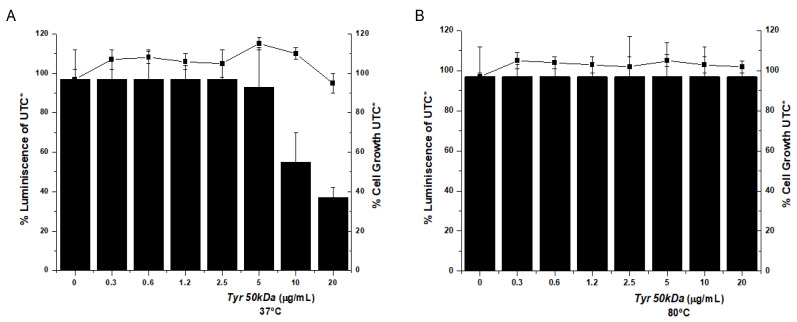
Inhibition of the hepatitis C virus (HCV) replicon in cell assays. Evaluation of the potency and cytotoxicity of temperature-inactivated Tyr50 kDa in cell (Huh-5-2) assays for (**A**) partially inactivated at 37 °C and (**B**) completely inactivated at 80 °C Tyr50 kDa. HCV replicon replication rate (luminescence, black bars) and cell survival (closed squares) were independently measured in cell culture by increasing protein concentration to determine EC50. * All luminescence and viability values were normalized considering the untreated control values (UTC).

To confirm the hypothesis that the inhibition of HCV replication is correlated to the tyrosinase activity, partial or complete inactive enzymes were prepared. Purified *Tyr50 kDa* was incubated at 37 °C or at 80 °C for 24 h. The catalytic activity involving L-DOPA as a substrate showed that incubation at 37 °C led to 80% reduction of activity, while incubation at 80 °C yielded a completely inactive enzyme. Then, the antiviral activity of these thermally treated enzymes was evaluated (Figure 7). Tyrosinase incubated at 37 °C gave an EC_50_ value of 10 µg/mL (10-fold lower inhibition than that of the fully active enzyme), while the *Tyr50 kDa* inactivated at 80 °C did not inhibit viral replication at all (Table 1). Therefore, these seem to indicate that the inhibitory mechanism of action of tyrosinase relies on its enzymatic activity.

**Figure 8:**
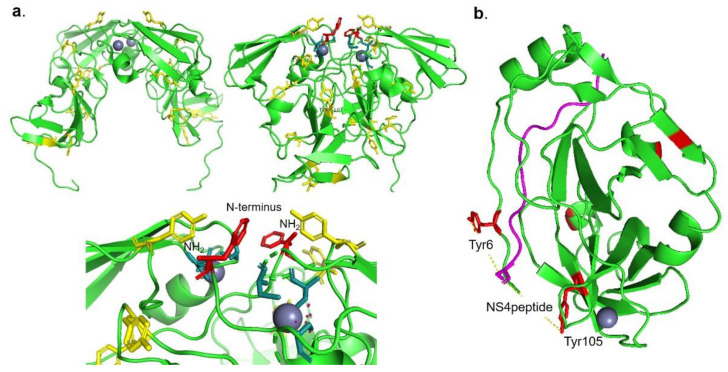
(**a**) 3D structure of the phosphoprotein NS5A, in dimeric form. Seven tyrosine residues (yellow) in each monomer, with one tyrosine in the N-terminal end (red). (**b**) 3D structure of the NS3 protease including the NS4 peptide (pink), showing the tyrosine residues in red. Structure was obtained from PDB with the following codes: 1zh1 (NS5) and 1jxp (NS3). Figures were drawn using Pymol program.

According to these results, a bioinformatic evaluation of the protein structures of NS3 and NS5A (Figure 8) showed that selective oxidation of tyrosine residues (Tyr93)—near to the active site—could be involved in the activity of dimer NS5A (Figure 8a) by potential imminent reaction with the amino terminal of the protein, thus precluding its location in the membrane and, therefore, blocking the replication of the virus. Tyr56 is also quite near to the catalytic active site for one possible inhibition of the enzymatic activity of the protease (Figure S5).

For example, in NS3 protein, Tyr 6 and Tyr105 are in a key position for the crucial NS3–NS4 interaction (Figure 8b), and where a possible oxidation of these groups could be affecting its interaction with NS4A, it is an instrument for blocking the replication of the virus.

For comparison, Ribavirin—a well-known drug for HCV, which is still used in combination with other drugs, such as Harvoni^®^, in the treatment of patients with cirrhosis—presented an EC_50_ of 10 µg/mL under the same conditions (Figure S7). This means that tyrosinase variant *Tyr50 kDa* was almost ten times more potent in vitro than ribavirin (Table 1).

Therefore, finally, after the demonstration that tyrosinases in mushrooms are responsible for antiviral activity, we proceeded to test the antiviral activity of enzymes’ mixture directly extracted from fresh *A. bisporus* common mushrooms.

By means of a simple extraction, together with solubilisation, precipitation, and dialysis steps, it was possible to obtain a purified protein sample of the two tyrosinases directly from fresh mushrooms. In this case, tyrosinases were obtained in their tetrameric form with all their subunits (Figure S6). This semi-purified tyrosinases solution (*TyrAB/Tyr50 kDa*-mushrooms) did not present cellular toxicity, giving a value for inhibition of viral replication similar to that obtained previously for the commercial extract, again corroborating its greater inhibitory capability of the virus in comparison with ribavirin (Table 2).

## 3. Materials and Methods

### 3.1. Materials

Tyrosinase from mushroom lyophilized powder (T3824-25KU), L-3,4-dihydroxy-phenylalanine (L-DOPA), Bradford’s reagent, and Triton™ X-100 were from Sigma-Aldrich (Spain). Octadecyl-Sepabeads^®^ (C18) was a gift from RESINDION, Mitsubishi Chemical Corporation.

### 3.2. In Vitro L-Dopa Activity Assay

Enzyme activity was tested in the presence of 2 mL of 1 mM L-DOPA in 0.1 M sodium phosphate buffer pH 7 at room temperature using V-730 spectrophotometer (Jasco), measuring the increase in absorbance of the aminochromes at 475 nm caused by 40 µL of enzyme solution, and taking the initial rate, between 10 and 70 s of the reaction. An enzyme activity unit (U) was defined as the amount of enzyme causing an increase of absorbance by 0.001/min at 25 °C.

Enzyme activity was tested varying the pH (acetate buffer at pH 5 and sodium phosphate buffer at pH 7), ionic strength of the medium (from 10 mM to 1 M of sodium phosphate buffer at pH 7), and detergent Triton™ X-100 (up to a concentration of 3%).

### 3.3. Purification of Commertial Extract for TyrAB and Tyr50 kDa Enzymes Solution

Here, 2 mL of tyrosinase extract solution in 100 mM buffer pH 7 (0.25 mg protein content calculated with Bradford protein assay of commercial lyophilized mushroom tyrosinase per mL of solution) was added to 0.5 g of octadecyl-Sepabeads^®^ and the mixture was incubated on a roller and the immobilization process was followed by a decrease in activity of the supernatant against L-DOPA in 30 min lapses up to 2 h, following the assay mentioned above. After that, solid containing both tyrosinases (C18-Tyrs) was washed abundantly with distilled water and filtrated by vacuum. Then, solid was incubated in Triton X-100 (0.25%) in water for 30 min for complete release of both tyrosinases and, after that, solution was recovered and stored at 4 °C. Solid support and supernatant were analyzed by SDS-PAGE. Finally, the soluble protein sample was purified to reduce the amount of detergent in the sample by ultrafiltration with Amicon Ultra 10 kDa (<0.05%).

### 3.4. Full Purification and Isolation of TyrAB and Tyr50 kDa

Here, 1 mg of commercial lyophilized tyrosinase was dissolved in 4 mL of distilled water. Then, 0.5 g of octadecyl-Sepabeads^®^ was added to this solution and the mixture was incubated for 2 h. After that, the mixture was vacuum filtered and the 4 mL supernatant was added to 1 mL solution of 500 mM sodium phosphate buffer at pH 7 containing 0.21% Triton X-100 (*w*/*v*) (to a final detergent concentration of 0.07%). Then, 0.5 g of fresh octadecyl-Sepabeads^®^ was added to this solution and the mixture was incubated for 3 h. After this time, the supernatant contained only the *Tyr50 kDa* enzyme, while *TyrAB* and a slight amount of *Tyr50 kDa* were on the solid (confirmed by SDS-PAGE) (Figure S4). *TyrAB* was recovered from the solid using a solution with detergent, as described in Section 3.3. Finally, the soluble protein samples were purified to reduce the amount of detergent in the sample by ultrafiltration with Amicon Ultra 10 kDa (<0.05%). The specific activity of the new purified tyrosinase 50 kDa was more than fourfold higher than the known *TyrAB.*

### 3.5. Tyrosinases Extraction Procedure Directly from the Mushroom

Here, 25 g of fresh white mushrooms (*A. bisporus*) (from Eroski supermarkets) were cut, selecting the cap and gills part, and then they were cut into small pieces and added to 50 mL of cold acetone. The mixture was kept under paddle stirring for 30 min in an ice bath. Then, the mixture was centrifuged at 7000 rpm for 20 min and the pellet was recovered, discarding the solution. This solid was suspended in 25 mL of distilled water and the mixture was incubated for 1 h, and then centrifuged at 8000 rpm for 40 min. After filtration, the supernatant was recovered and the saline precipitation process was performed, adding 60% (*w*/*v*) of ammonium sulfate, adding it little by little, followed by 1 h of incubation. After that, the mixture was centrifuged at 8000 rpm for 40 min and the solid was recovered and stored at −20 °C. Solid was dissolved in water and dialysis was performed before use. SDS-PAGE analysis was conducted (Figure S6).

### 3.6. Evaluation of Cellular Toxicity and Antiviral Activity

The highly permissive cell clone Huh 7-Lunet, as well as Huh 7 cells containing subgenomic (HCV) replicons I389luc-ubi-neo/NS3-3′/5.1 (Huh 5–2), I377NS3-3′/wt (Huh 9–13), or I389/hygro-ubi-NS3-3/5.1 (a kind gift from Dr. V. Lohmann and Dr. R. Bartenschlager), have been previously described [31]. Briefly, this system allowed the efficient propagation of genetically modified HCV RNAs (replicons) in a human hepatoma cell line (Huh). The amount of RNA that has been transcribed and translated is determined through the quantification of a reporter contained in the replicon system (luciferase). The amount of luminescence detected (after adding the substrate specific for this enzyme) is proportional to the virus replication rate [31]. Cells were grown in Dulbecco’s modified Eagle’s medium (DMEM) supplemented with 10% heat-inactivated fetal bovine serum, 1 × non-essential amino acids, 100 IU mL^–1^ penicillin, 100 μg mL^–1^ streptomycin, and 250 μg mL^–1^ geneticin (G418).

Huh-5-2 (Lunet, liver tumor cells) cells were transfected with a plasmid containing a replicon system (pseudovirus including all the non-structural proteins from the hepatitis C virus: NS2, NS3, NS4A, NS4B, NS5A, and NS5B). Because the replicon system does not contain structural proteins, it is not infective; however, it replicates autonomously and allows quantifying, through a reporter gene encoding for the enzyme luciferase, the rate of replication within the cells is proportional to the luminescence signal after adding an appropriate luciferase substrate. In the case of inhibition of replication, a reduction of the luminescence signal will be observed compared with the corresponding control.

The Huh5-2 cell culture was performed using DMEM with phenol red as the culture medium, supplemented with fetal bovine serum (FBS), penicillin, glutamine, streptavidin, non-essential amino acid, and 500 µg/mL geneticin (G418) from Invitrogen (selection of cells with a replicon system inside them). The culture conditions were as follows: temperature (37 °C), 5% CO_2_, 95% air, and 1 atm of pressure.

To determine both the antiviral activity of tyrosinases and their cellular toxicity, 96-well plates were seeded with 7000 cells/well (using DMEM without phenol red) with 100 μL final volume, in which increasing serial concentrations of tyrosinases (from 0 to 0.2 mg/mL) were tested in triplicate for each of the concentrations. The cells were incubated for 72 h with tyrosinases, after which both their cytotoxicity and their antiviral activity were evaluated (in duplicate plates).

For cytotoxicity, in one of the plates, the cellular metabolism level of each of the wells was determined using the Cell Titer 96^®^AQueous Reagent (Promega) in an absorbance plate reader (Synergy HT Multi-Modal Microplate Reader with Gen5 Data Analysis software, BioTek). The supernatant from the wells was removed, and 20 µL of Cell Titer 96^®^AQueous was diluted 1:4 in the same DMEM culture medium, but with no phenol red added, and incubated for 3 h at 37 °C. The absorbance was measured at 490 nm and 800 nm, and the difference between these absorbance values reports the substrate metabolized by viable cells in the culture. Each tyrosinase concentration was evaluated in triplicate, and the values obtained were averaged. The relationship between the tyrosinase concentration and the percentage of absorbance was plotted (taking as 100% those values in the control wells with no tyrosinase treatment). The lethal concentration 50% (LC50) was calculated as the tyrosinase concentration at which cell viability was reduced by 50% (with respect to that obtained in the absence of tyrosinase treatment).

For antiviral activity, the luminescence of each of the wells was determined using the Bright-Glo™ Luciferase Assay System Reagent (Promega) in a luminescence plate reader (Synergy HT Multi-Modal Microplate Reader with Gen5 Data Analysis software, BioTek). Without removing the supernatant, 30 µL of reagent was added to each well, and the luminescence signal was measured. The luminescence signal was proportional to the amount of the transcribed reporter gene. Each tyrosinase concentration was evaluated in triplicate, and the values obtained were averaged. The relationship between the concentration of tyrosinase and the percentage of luminescence was plotted on a graph (taking as 100% those values in the control wells with no tyrosinase treatment). EC50 was calculated as the tyrosinase concentration at which viral replication was reduced by 50% (with respect to that obtained in the absence of tyrosinase treatment).

The assay was made using the same procedure as described above for tyrosinase, but using ribavirin (Figure S7).

## 4. Conclusions

Two tyrosinases (a well-known enzyme *TyrAB* plus a new 50 kDa tyrosinase isoform, *Tyr50 kDa*) present in white common mushrooms (*A. bisporus*) have shown antiviral activity against the hepatitis C virus. After isolating both purified enzymes, we have demonstrated that both enzymes are the unique active molecules in the antiviral activity of the protein extract. Furthermore, this inhibitory activity seems to be directly correlated to the enzymatic activity of the tyrosinases, which seems to indicate, for the first time, a new mechanism of action compared with other pharmaceuticals (e.g., such as Rivabirin). This tyrosinases solution showed ten times higher in vitro antiviral activity than Ribavirin.

Therefore, this protein preparation might become a promising therapeutic agent, providing a low-cost drug for the treatment of hepatitis C, which could be used as a substitute or in combination with other drugs, such as, e.g., Sofosbuvir in Harvoni^®^.

This new viral inhibition mechanism of action discovered for tyrosinase could be a promising broad spectrum pharmacological agent (e.g., effective for other virus).

## Figures and Tables

**Table 1:** EC_50_ and LC_50_ of the different isoforms of tyrosinase purified from the commercial extract of *A. bisporus*.

Entry	Protein	EC_50_ (µg/mL)	LC_50_ (µg/mL)
1	*TyrAB*	6–12	ND (>>25)
2	*Tyr50 kDa*	1.2–2.5	ND (>>25)
3	*Tyr50 kDa* (37 °C) ^1^	10	ND (>>25)
4	*Tyr50 kDa* (80 °C) ^2^	ND (>20)	ND (>>25)
12	Rivabirin	10	ND (>>20)

^1^ Partially inactivated enzyme; ^2^ fully inactivated enzyme; ND: not determined at the concentrations tested.

**Table 2:** EC_50_ and LC_50_ of the enzyme solution obtained directly from mushroom compared with ribavirin.

Entry	Sample	EC_50_ (µg/mL)	LC_50_ (µg/mL)
1	*TyrAB/Tyr50 kDa-mushrooms*	2.5	10
2	Ribavirin	10	ND (>>20)

ND: not determined at the concentrations tested.

## Data Availability

Data is contained within the article and supplementary material.

## Supplementary Materials

The following are available online at https://www.mdpi.com/article/10.3390/ph14080759/s1, Figure S1: SDS-PAGE with silver staining of the commercial extract of *A. bisporus* from Sigma, Figure S2: *Agaricus bisporus* tyrosinase surface structure, Figure S3: SDS-PAGE- coomassie staining analysis of the two-step chromatography purification cascade, Figure S4: SDS-PAGE analysis of the two-step chromatography purification process for *TyrAB* and *Tyr50* kDa, Figure S5: 3D structure of the active site area of phosphoprotein NS5A, Figure S6: SDS-PAGE with coomassie staining of the enzymes solution directly produced from fresh mushrooms, Figure S7: Inhibition of the Hepatitis C virus (HCV) replicon in cell assays.
