# Supplementary material for: In Vitro Antiviral Activity of Tyrosinase from Mushroom Agaricus bisporus against Hepatitis C Virus
Source: Pharmaceuticals (Basel). 2021 Aug 3;14(8):759. doi: 10.3390/ph14080759 (PMC8399381; doi:10.3390/ph14080759)
Supplement: Supplementary file 1 [file pharmaceuticals-14-00759-s001.zip › pharmaceuticals-1288063-supplementary.pdf]

Supporting Information

In vitro antiviral activity of tyrosinase from mushroom *Agaricus bisporus* against hepatitis C virus

David Lopez-Tejedor,[1] Rafael Clavería-Gimeno,[2-6] Adrian Velazquez-Campoy,[3-7] Olga Abian[2-6]* and Jose M. Palomo[1,]*

Methods

SDS-PAGE

12% separating gel and 4% stacking gel were used for SDS-PAGE. The samples were heated at 100 ºC for 5 minutes (at least) in Eppendorf tubes in 1:1 ratio with 2X loading buffer (4% w/v of SDS, 8% v/v of glycerol, 4 mM of beta-mercaptoethanol, 80 mM Tris-HCl pH 6.8 and 0.02% of Bromophenol Blue) and the electrophoresis was performed in PerfectBlue™ Dual Gel Twin S (PEQLAB) with power supplier PS 300-B (AlphaMetrix) at 185 V for 72 minutes in Tris-HCl buffer pH 8.3. After that, Coomassie blue (G-250) and silver staining were used. GelAnalyzer2010 software was used for detection and quantification of protein bands.

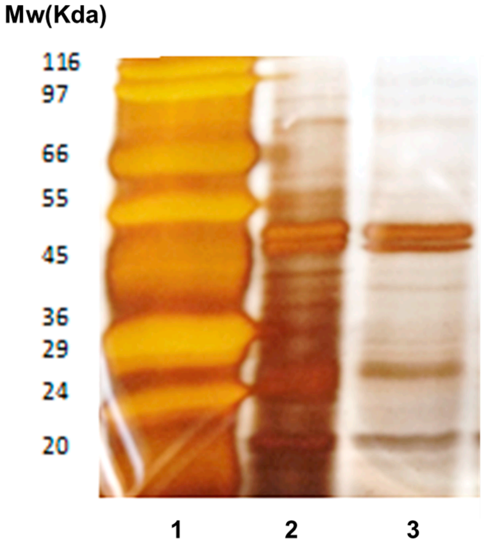

Figure S1. SDS-PAGE with silver staining of the commercial extract of *A. bisporus* from Sigma. Lane 1: molecular marker. Lane 2. Tyrosinase extract from Sigma 0.25 mg protein/mL. Lane 3. Tyrosinase extract from Sigma 0.1 mg protein/mL.

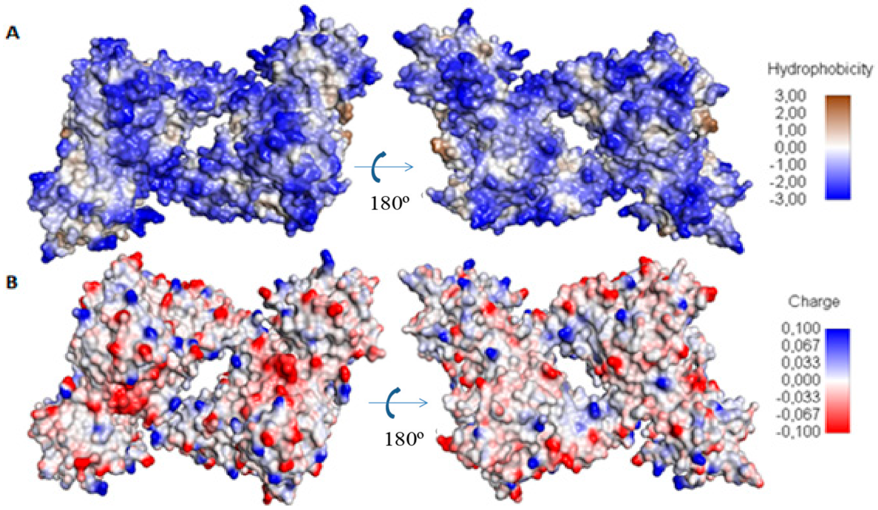

Figure S1. *Agaricus bisporus* tyrosinase surface structure. **A**) Hydrophobicity distribution, B) Charge distribution, front and back (PDB 2Y9W).

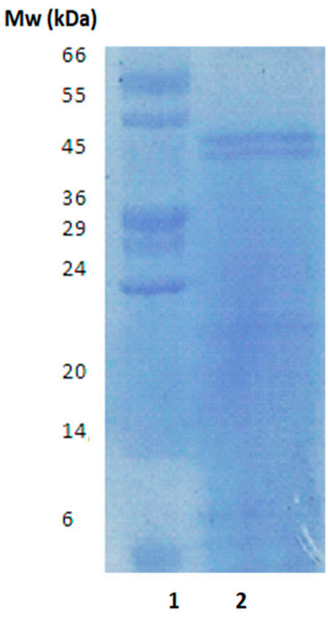

Figure S3. SDS-PAGE- coomassie staining analysis of the two-step chromatography purification cascade. Lane 1 – Molecular Weight marker. Lane 2- supernatant of C18 adsorption at 100 mM buffer pH 7 of tyrosine extract after desorption with 0.2 % triton X-100.

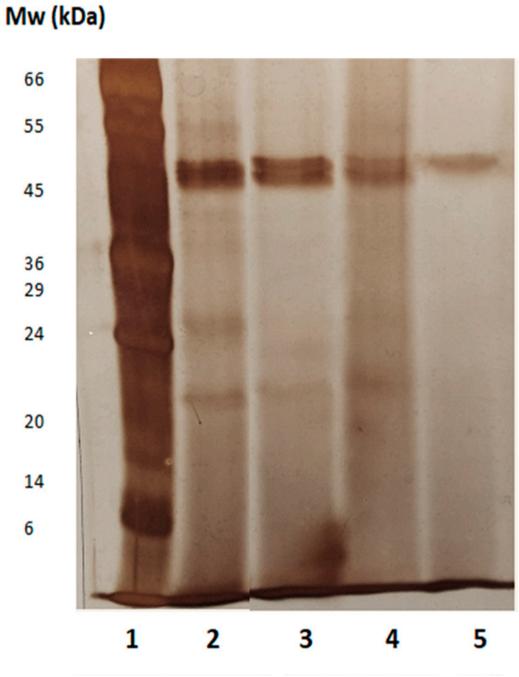

Figure S4. SDS-PAGE analysis of the two-step chromatography purification process for *TyrAB* and *Tyr50kDa*. Lane 1 – Molecular Weight marker. Lane 2 – tyrosinase extract from Sigma. Lane 3 – supernatant of C18 adsorption in distilled water. Lane 4 –supernatant on C18 support at 100 mM buffer +0.07% triton solution. Lane 6 – supernatant after C18 (lane 5) immobilization (only Tyr50kDa remaining).

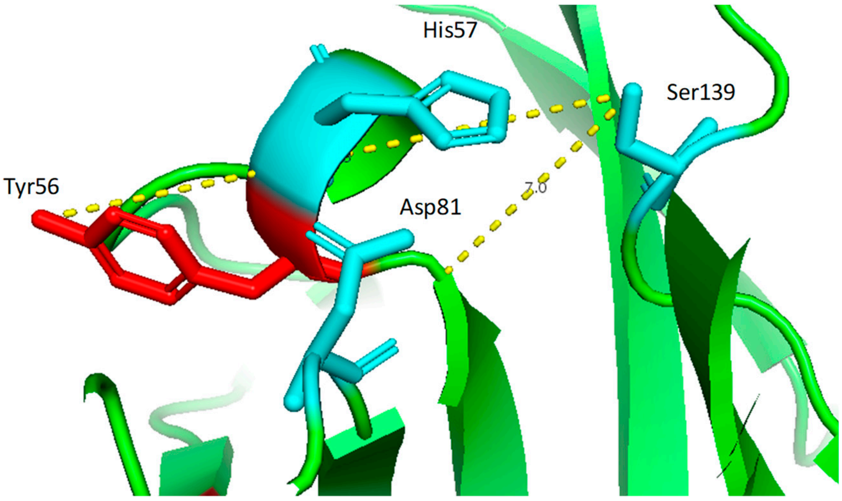

Figure S5. 3D structure of the active site area of phosphoprotein NS5A. Active site amino acids (blue), Tyr56 (red). Structure was obtained from PDB data bank with following codes: 1zh1. Figure was drawn using Pymol program.

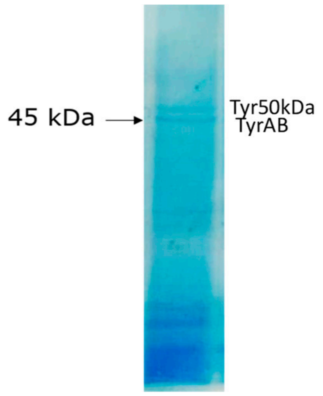

Figure S6. SDS-PAGE with coomassie staining of the enzymes solution directly produced from fresh mushrooms. Lane 1: Tyrosinases solution.

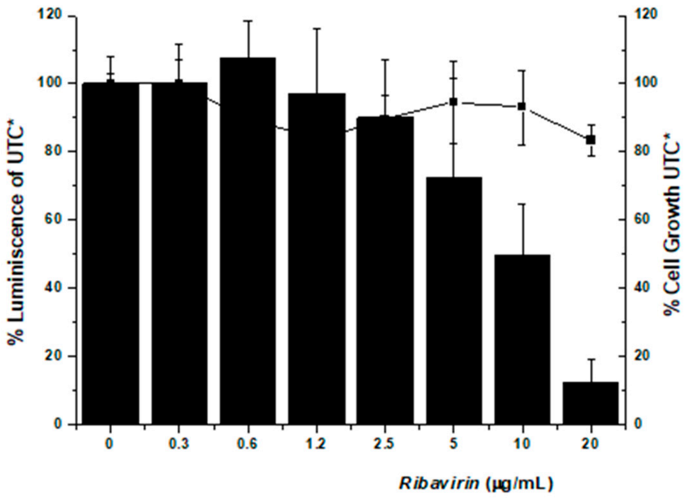

Figure S7. Inhibition of the Hepatitis C virus (HCV) replicon in cell assays. Evaluation of the potency and cytotoxicity of Ribavirin in cell (Huh-5-2-) assays. HCV replicon replication rate (luminescence, black bars) and cell survival (closed squares) were independently measured in cell culture by increasing protein concentration to determine EC50. *All luminescence and viability values have been normalized considering the untreated control values (UTC).
